# Propensity score matching assessment of direct medical charges for patients with healthcare-associated infections

**DOI:** 10.3389/fpubh.2026.1819607

**Published:** 2026-06-01

**Authors:** Xinhai Zhao, Kuiqing Lu, Qiangsheng He, Yan Lu, Huiping Huang, Youpeng Chen

**Affiliations:** 1 Hospital Infection Management Department, The Seventh Affiliated Hospital, Sun Yat-sen University, Guangzhou, Guangdong, China; 2 Clinical Research Center, Seventh Affiliated Hospital, Sun Yat-sen University, Guangzhou, Guangdong, China; 3 Department of Infectious Diseases, The Seventh Affiliated Hospital, Sun Yat-sen University, Guangzhou, Guangdong, China

**Keywords:** diagnosis-related groups, direct medical costs, healthcare-associated infections, patient-payer perspective, propensity score matching

## Abstract

**Importance:**

Healthcare-associated infections (HAIs) represent a significant source of harm related to healthcare delivery and are associated with substantial costs. A more precise estimation of the economic burden imposed by these infections can assist healthcare providers and payers in justifying investments in prevention strategies.

**Objective:**

To quantify the direct economic burden of healthcare-associated infections and identify the specific types of HAIs that impose the greatest economic costs.

**Methods:**

This is a retrospective case–control study. We enrolled all inpatients with hospitalizations lasting three calendar days or longer (from January 2023 to December 2024) at a Grade A tertiary hospital in Shenzhen, China, and propensity score matching (PSM) was applied for analysis. Demographic/clinical characteristics and direct medical costs were extracted from the EMR-integrated data analytics platform. In contrast, HAI surveillance data and laboratory parameters were sourced from the real-time HAI monitoring system.

**Results:**

Following PSM, the HAI group had significantly higher median total hospitalization costs than the non-HAI group (USD 7,158 vs. USD 1,212; *p* < 0.001), with all 11 cost components differing—pharmaceuticals (USD 1,650 (95% CI: 1457, 1891)) and lab tests (USD 928 (95% CI: 859, 1,042)) as primary contributors. When stratified by HAI type, LRTIs were the most prevalent and exhibited the higher total charges difference (USD 9,597; 95% CI: 8,099 to 10,968). This difference was primarily driven by higher median charges for pharmaceuticals (USD 2,698; 95% CI: 1,019 to 7,156) and laboratory tests (USD 1,574; 95% CI: 776 to 3,194). Among all infection categories, CLABSIs were associated with the highest increase in laboratory test charges.

**Conclusion:**

The HAI group incurred higher total hospitalization charges compared to the non-HAI group, with increases observed across all individual charge components, including pharmaceuticals and laboratory test charges, which were the primary drivers of the overall increase. Among the various types of HAIs, CLABSIs resulted in the highest laboratory charges, while LRTIs were identified as the most prevalent and demonstrated significantly increased direct medical charges.

## Introduction

Healthcare-associated infections (HAIs) are defined as infections acquired by patients during hospitalization, encompassing both those manifesting during inpatient stay and those identified post-discharge that originated within the healthcare facility. These exclude infections with onset before admission or present at the time of admission.

The implementation of HAI control programs necessitates substantial investments in human, material, and financial resources. However, a prevalent misconception among many Chinese medical practitioners and even some hospital administrators is that infection control constitutes a pure cost burden without measurable returns. This perspective overlooks that, while HAI prevention involves upfront investment, it reduces morbidity and mortality, shortens hospital stays, and limits the development of antimicrobial resistance. These effects underscore the evidence-based clinical and economic value of HAI prevention, positioning it as a high-impact strategy that improves patient outcomes and optimizes healthcare system efficiency.

In reality, healthcare-associated infection (HAI) control not only alleviates patient suffering and enhances care quality but also reduces hospital length of stay (LOS), increases bed turnover rates, and lowers patients’ medical expenditures. However, while controlling healthcare-associated infections is known to alleviate patient suffering and reduce hospital costs, there is limited research quantifying the specific costs attributable to HAIs from the patient payment perspective.

While HAIs unequivocally increase medical costs for affected patients, the exact drivers of these cost increments lack consensus. A study on the direct economic burden of HAIs in cancer patients demonstrated increased direct medical costs and prolonged LOS, with heterogeneity across tumor types, infection sites, and age groups (1). For instance, research from Chinese tertiary public hospitals quantified an additional per capita expenditure of USD 2,047 for HAI patients versus non-HAI controls, of which 50% was attributable to pharmaceutical costs (2).

Our study employed propensity score matching (PSM) to analyze hospitalized patients with hospital stays longer than three calendar days between January 2023 and December 2024. The objective of this study was to quantify the incremental direct medical charges attributable to healthcare-associated infections (HAIs), thereby providing empirical evidence for health economic assessments of HAI-related financial burdens.

## Materials and methods

### Research design

This study employed a retrospective case–control design, enrolling all inpatients whose hospitalization duration exceeded three calendar days between January 2023 and December 2024. The investigation was conducted at a Level 3 Grade A tertiary teaching hospital in Shenzhen, China—a designated regional referral center specializing in the management of complex and critical care, with a focus on Gastrointestinal Surgery. From 2023 to 2024, the hospital admitted an average of 40,000 patients annually and reported an average healthcare-associated infection incidence of 1.5%.

**Figure 1:**
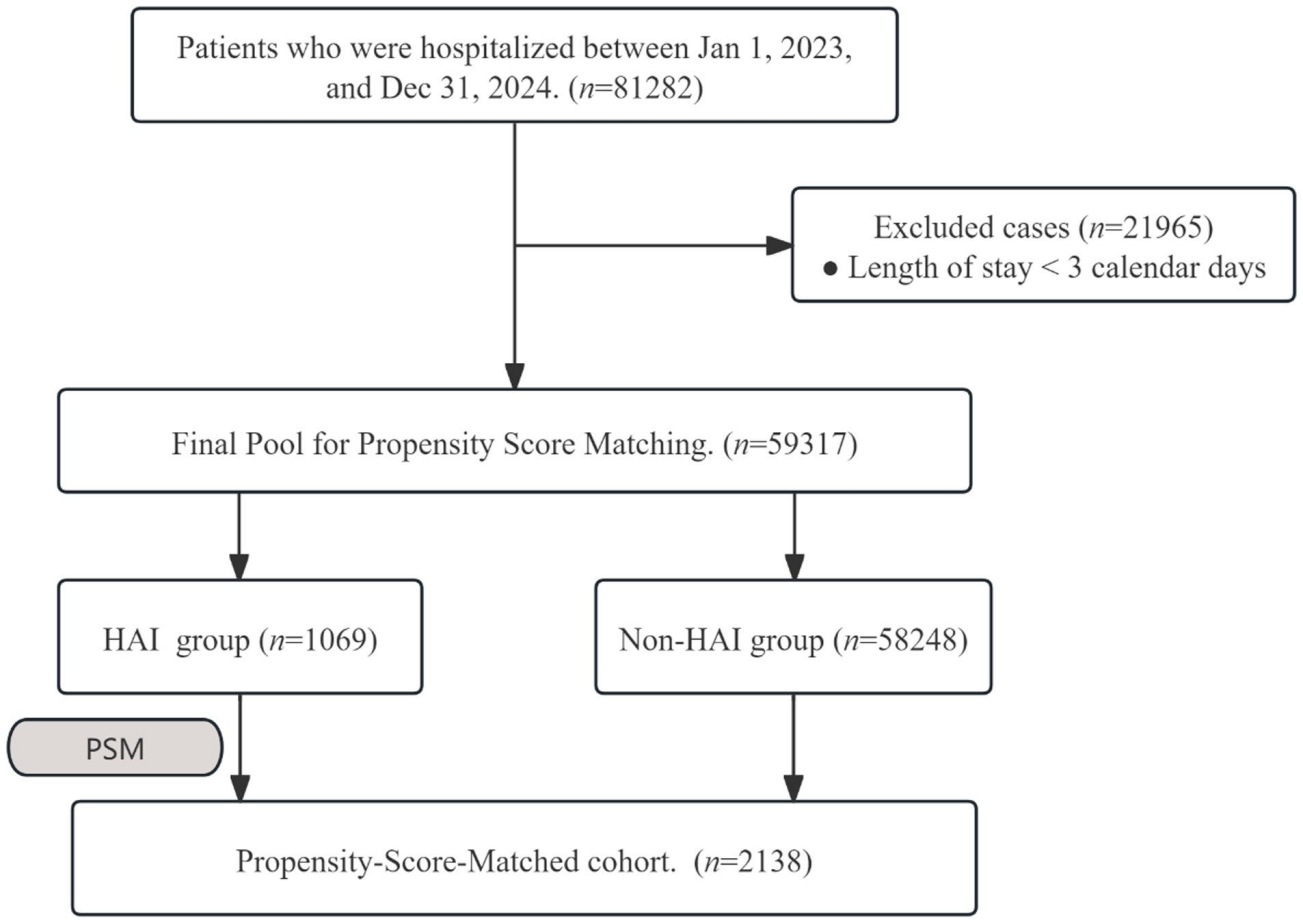
Flowchart of the propensity-score-matched cohort for healthcare-associated infections among inpatients, 2023 -2024.

Demographic characteristics, clinical diagnoses, and direct medical payment records were extracted from the hospital’s data analytic platform interfaced with the electronic medical record (EMR) system. HAI surveillance data and laboratory parameters were extracted from the hospital’s real-time HAI surveillance system (see Figure 1).

### Diagnostic criteria and definitions for healthcare-associated infections

The diagnostic criteria were based on the Healthcare-Associated Infection Diagnostic Standards issued by the Ministry of Health of China in 2001 (3). With reference to the general principles outlined in the 2025 guidelines for healthcare-associated infections (4), Healthcare-associated infections refer to infections that occur in patients during their stay in a healthcare facility. This includes infections that first appear during the hospitalization itself, as well as those with an onset after discharge that are determined to have been acquired within the hospital environment. This standard excludes cases in which infections existed before admission or at the time of admission. All potential HAI cases flagged by the real-time surveillance system were verified. Infection control professionals were responsible for assessing the alerts and confirming the final diagnosis.

### Case and control groups

All hospitalized patients who met the HAI diagnostic criteria were selected as cases. Using propensity score matching (PSM), non-HAI patients were successfully matched and assigned to the control group.

### Medical charges calculation

For each patient, the total hospitalization charges incurred from the third day of admission onward were retrieved via the hospital’s big data analytics platform for clinical services, establishing the third day as the uniform starting point for charge analysis to avoid potential bias from expenses during the initial three-day period. The data extracted from the big data system for clinical services represented the gross charges before any refunds or discounts, reflecting nominal direct medical charges that patients paid, converted to USD, and were not derived from accounting-based cost estimations.

The cost items are broken down as follows: consultation charges, diagnostic test charges, non-pharmacological therapy charges, surgical procedure charges, laboratory test charges, nursing service charges, other charges, medical supply charges, blood transfusion charges, bed charges, pharmaceutical charges, Chinese patent medicine charges, and Chinese herbal medicine charges. Among these charges, total medication charges refer to the sum of pharmaceutical charges, Chinese patent medicine charges, and Chinese herbal medicine charges. Total hospitalization charges represent the aggregate of all expenses incurred during hospitalization, including consultation charges, diagnostic test charges, non-pharmacological therapy charges, surgical procedure charges, laboratory test charges, nursing service charges, other charges, and total medication charges.

Traditional Chinese medicines (TCMs) are formulations derived from raw or processed botanical parts (e.g., leaves, buds, flowers, stems, roots, or tubers), minerals (such as borneol), and/or animal products (e.g., prepared scorpions or earthworms). They are categorized into Chinese herbal medicines (5) and Chinese patent medicines (6). In China, certain TCMs are considered to possess antibacterial and anti-inflammatory properties and are sometimes prescribed by physicians to patients with healthcare-associated infections. Although these properties have been reported in previous studies (5, 6), their precise mechanisms of action remain unclear.

### Confounding factor

In analyzing factors influencing hospitalization expenditures related to healthcare-associated infections, our study incorporated the following variables, drawing on previous literature: admission date (7); age; sex; admitted ward; malignant tumor (8); obesity (9); diabetes mellitus (10); chronic kidney disease (11); hypertension (12); cardiovascular disease (CVD) (13); chronic obstructive pulmonary disease (COPD) (14); and whether any surgical procedure was performed during hospitalization or the pre-infection period. Surgical procedures were defined based on data extracted from the electronic medical records and encompassed all operative interventions documented during index hospitalization. Case severity was adjusted using the DRG weight, a relative weight (RW) that reflects the average resource intensity of a given diagnosis group.

Notably, the length of stay was not used as a matching variable. This decision was based on the rationale that patients who develop infections inherently have an increased length of stay, which is not only associated with infection but also contributes to elevated bed charges and total hospitalization charges, with length of stay functioning as an intermediate variable. Matching on this variable may lead to overmatching.

### Information collection

Data collection was performed in two steps. First, data on the total hospitalization charges of all patients discharged between 2023 and 2024 were retrieved from the hospital’s data analytics platform. Concurrently, data on patients with healthcare-associated infections during the same period were obtained from the real-time HAI surveillance system. These two datasets were then merged via the unique patient hospitalization identifier to create the initial analytical dataset.

### Statistical analysis

In observational studies comparing two distinct populations with different baseline characteristics or disease progression rates, inherent imbalances in potential confounding factors may introduce bias into the results. While randomized controlled trials mitigate such bias through the random allocation of interventions, observational studies, including real-world studies, do not allow for the random assignment of subjects. Therefore, statistical methods are necessary to balance confounding factors between groups. PSM matches individuals from the two cohorts on the basis of selected covariates, thereby helping to mitigate potential confounding (15).

All the statistical analyses, including descriptive statistics, normality testing, bootstrap, and PSM, were performed via R software (version 4.5.1). Propensity scores for matching were estimated using a generalized linear model with a logit link function. A 1:1 caliper-restricted nearest-neighbor matching method without replacement was applied based on the logit of the propensity score, with a caliper width set to 0.02. Between-group comparisons of continuous variables were conducted using the Mann–Whitney *U* test. Data visualization was performed via GraphPad Prism (version 10.4). The level of statistical significance was set at *p* < 0.05.

## Results

Data from 81,262 patients discharged between January 2023 and December 2024 were initially extracted. After excluding 21,965 patients whose length of stay was less than 3 calendar days, 59,317 patients remained for analysis. This final cohort included 1,069 patients with HAIs and 58,248 non-HAIs.

### Propensity score matching

**Figure 2:**
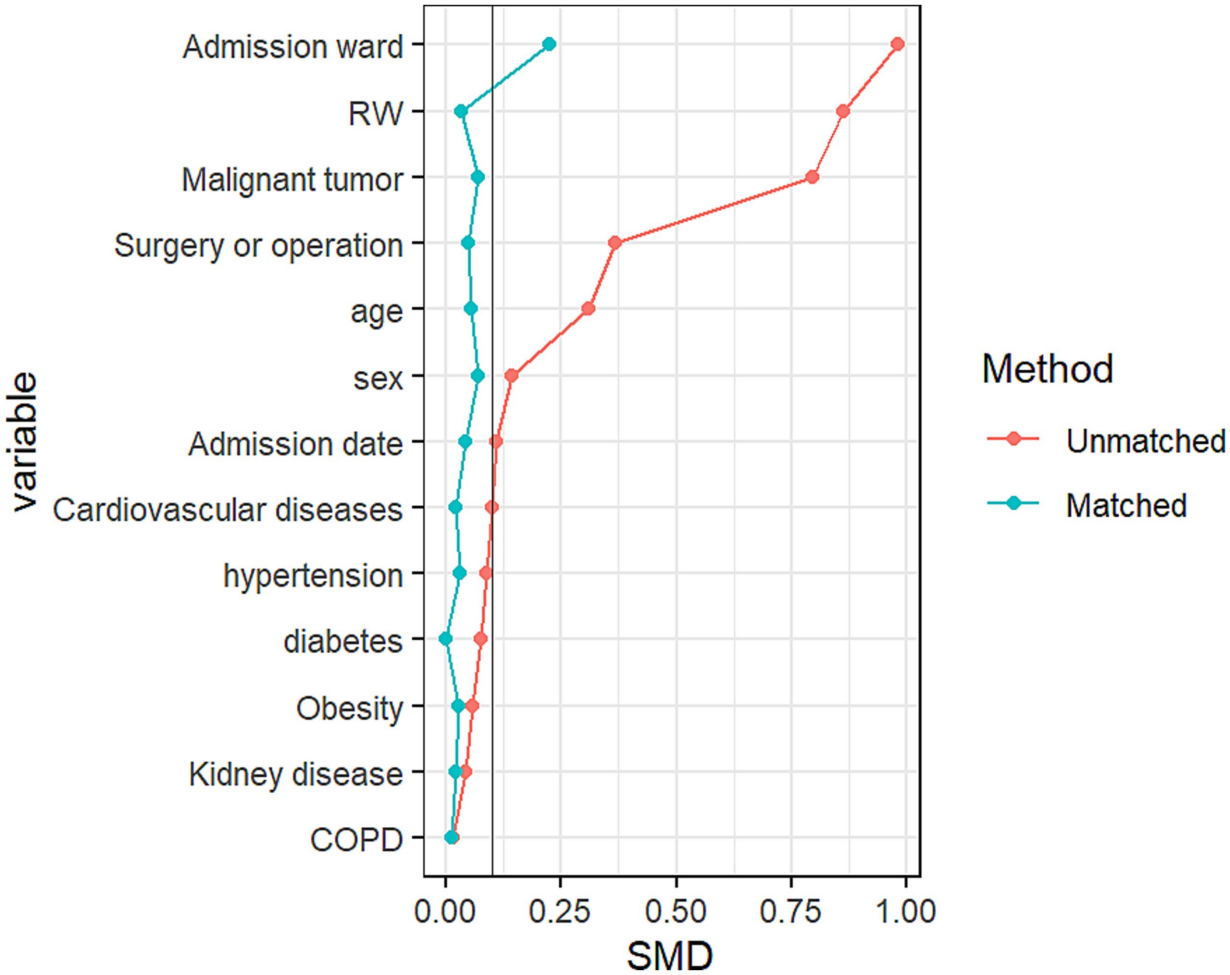
Distribution of the SMD of each covariate before and after PSM matching.

**Figure 3:**
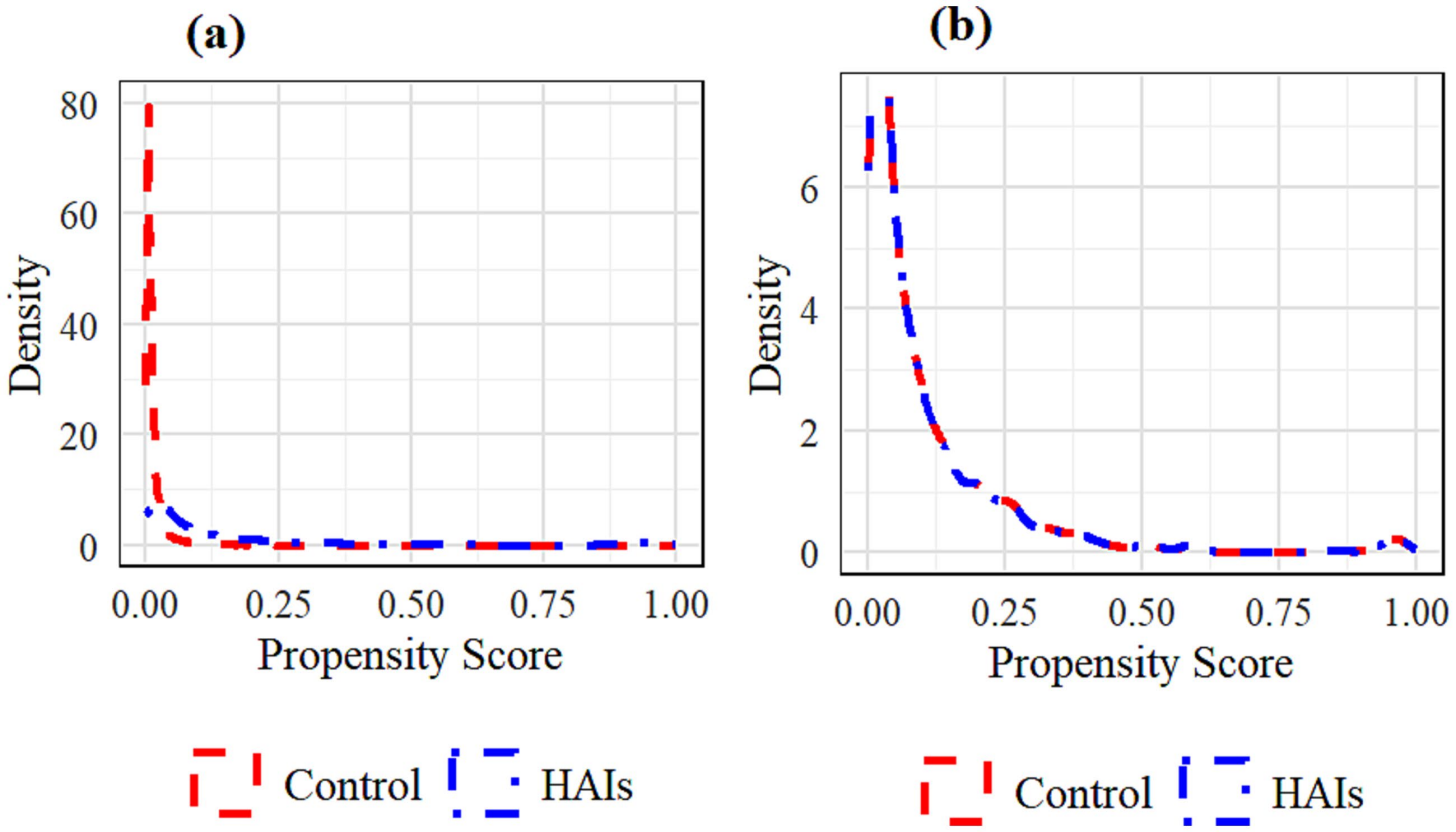
Propensity score distributions before and after matching: **(a)** Before matching and **(b)** after matching.

Propensity score matching was performed using the following covariates: admission date, RW, age, sex, admission ward, cancer status, obesity status, diabetes mellitus status, chronic kidney disease, hypertension status, cardiovascular disease, chronic obstructive pulmonary disease, whether a surgical procedure was performed during hospitalization, To facilitate the matching algorithm, the admission date was converted to a numerical variable using a baseline date of January 1, 2020. Before matching, admission ward, RW, malignant tumor, surgery or operation, age, sex and admission date, cardiovascular diseases demonstrated imbalance, with standardized mean differences (SMDs) greater than 0.1. After PSM, the balance of covariates substantially improved. All covariates achieved good balance (SMD < 0.1), except for admission ward, which retained an SMD slightly above this threshold. We conducted a sensitivity analysis by varying the matching caliper to assess the robustness of the PSM covariate balance. Based on the results of this analysis, we selected a caliper of 0.02 for the primary analysis, see Table 1. Among the initial 1,137 patients with HAI, 1,069 (94.0%) were successfully matched. The distributions of the SMD before and after matching are presented in Figure 2 and Table 2. The density graphs before and after PSM show that the PSM score became more balanced after matching, as Figure 3 shows.

**Table 1:** Assessment of covariate balance under different calipers.

Caliper	HAI/non-HAI (*n*)	Pre-/post-matching composition (%)	Variables with SMD > 0.1 after matching
None	1137/1137	100	Admission ward
0.1	1091/1091	95.95	Admission ward
0.02^*^	1069/1069	94.02	Admission ward
0.001	973/973	85.68	Admission ward, RW

*Caliper = 0.02 was selected as the final matching parameter for the primary analysis.

**Table 2:** Distribution of covariates before and after PSM.

Covariate	Unmatched			Matched		
	Non-HAI (*n* = 58,248)	HAIs (*n* = 1,137)	SMD	Non-HAI (*n* = 1,069)	HAIs (*n* = 1,069)	SMD
Admission date (*M* ± SD)	1479.80 ± 204.49	1457.69 ± 201.20	0.109	1457.11 ± 203.56	1457.47 ± 201.29	0.002
Sex (%)			0.145			0.030
Female	28,670 (49.2)	478 (42.0)		468 (43.8)	452 (42.3)	
Male	29,578 (50.8)	659 (58.0)		601 (56.2)	617 (57.7)	
Age (*M* ± SD)	45.68 ± 20.36	52.11 ± 21.32	0.309	53.39 ± 19.93	52.39 ± 21.40	0.048
Admission ward			0.979			0.195
Malignant tumor cancer (%)			0.794			0.065
No	43,875 (75.3)	441 (38.8)		399 (37.3)	433 (40.5)	
Yes	14,373 (24.7)	696 (61.2)		670 (62.7)	636 (59.5)	
Obesity (%)			0.058			<0.001
No	57,372 (98.5)	1,127 (99.1)		1,059 (99.1)	1,059 (99.1)	
Yes	876 (1.5)	10 (0.9)		10 (0.9)	10 (0.9)	
Diabetes (%)			0.070			0.009
No	47,996 (82.4)	903 (79.4)		837 (78.3)	841 (78.7)	
Yes	10,252 (17.6)	234 (20.6)		232 (21.7)	228 (21.3)	
Kidney disease (%)			0.044			0.004
No	55,905 (96.0)	1,081 (95.1)		1,015 (94.9)	1,016 (95.0)	
Yes	2,343 (4.0)	56 (4.9)		54 (5.1)	53 (5.0)	
Hypertension (%)			0.088			0.006
No	43,420 (74.5)	803 (70.6)		751 (70.3)	754 (70.5)	
Yes	14,828 (25.5)	334 (29.4)		318 (29.7)	315 (29.5)	
Cardiovascular diseases (%)			0.102			0.002
No	45,275 (77.7)	834 (73.4)		778 (72.8)	779 (72.9)	
Yes	12,973 (22.3)	303 (26.6)		291 (27.2)	290 (27.1)	
COPD (%)			0.017			0.032
No	57,810 (99.2)	1,130 (99.4)		1,059 (99.1)	1,062 (99.3)	
Yes	438 (0.8)	7 (0.6)		10 (0.9)	7 (0.7)	
Surgery or operation (%)			0.367			0.021
No	40,818 (70.1)	597 (52.5)		565 (52.9)	576 (53.9)	
Yes	17,430 (29.9)	540 (47.5)		504 (47.1)	493 (46.1)	
RW (*M* ± SD)	1.13 ± 1.1	3.65 ± 3.98	0.863	2.76 ± 3.37	3.02 ± 3.05	0.080

### The difference in hospitalization charges for HAIs

Successful propensity score matching revealed a significant absolute difference in median total hospitalization costs of USD 5,946(95%CI: 5295, 6,835) between patients with and without healthcare-associated infections (see Table 3).

**Table 3:** Hospitalization expenses for infected and non-infected patients after PSM.

Charges	HAIs group			Non-HAI group			Difference (95% CI)
	P_25_	*M*	P_75_	P_25_	*M*	P_75_	
Consultation charges	33.70	109.5	232.09	0	17.03	76.63	92.47 (78.96, 106.18)
Diagnostic test charges	107.4	407.1	1266.00	0	25.56	364.7	381.54 (328.82, 438.25)
Non-pharmacological therapeutic charges	164.80	514.50	1425.00	4.83	51.93	325.30	462.57 (406.51, 527.13)
Surgical procedure charges	0	24.83	746.40	0	0	283.30	24.83 (10.53, 36.19)
Laboratory test charges	427.80	1028.00	2288.00	2.40	99.70	654.90	928.30 (859.55, 1042.99)
Nursing service charges	64.85	161.10	374.90	3.37	22.19	79.62	138.91 (125.18, 156.5)
Other charges	0	0	0	0	0	0	0 (0, 0)
Medical supply charges	12.92	236.50	1684.00	0	8.99	668.20	227.51 (172.3, 290.53)
Blood transfusion charges	0	0	0	0	0	0	0 (0, 49.85)
Bed charges	85.43	174.00	373.10	12.20	42.27	108.6	131.73 (120.2, 149.71)
Pharmaceutical charges	619.50	1858.00	4476.00	16.88	207.20	988.20	1650.80 (1445.4, 1891.86)
Chinese patent medicine charges	0	0.44	16.08	0	0	6.23	0.44 (0, 1.7)
Chinese herbal medicine charges	0	0	0	0	0	0	0 (0, 0)
Total pharmaceutical charges	632.60	1875.00	4533.00	23.12	220.90	1026.00	1654.10 (1457.49, 1891.75)
Total hospitalization charges	2553.00	7158.00	14682.00	109.40	1212.00	5522.00	5946.00 (5295.52, 6835.35)

**Figure 4:**
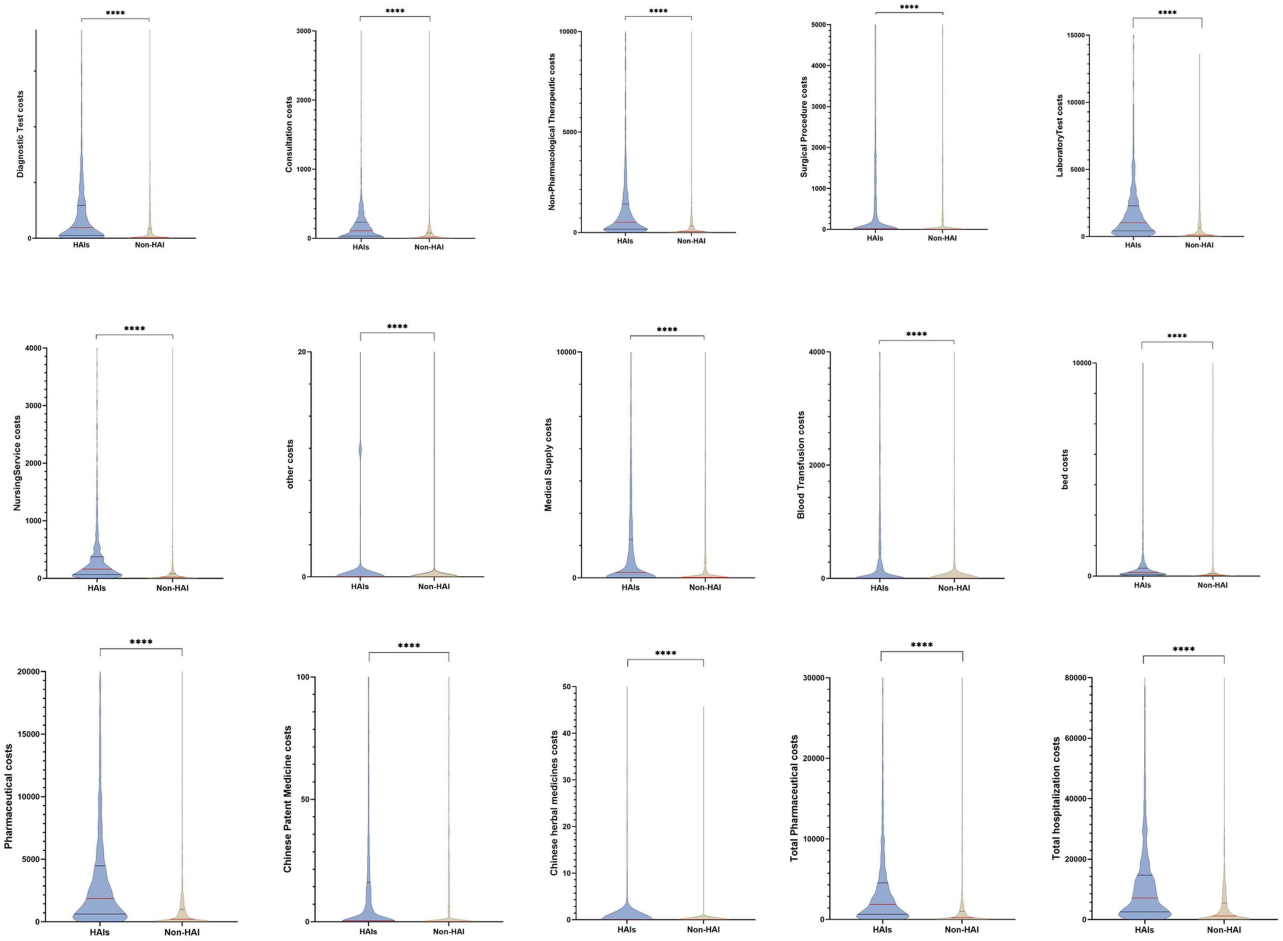
Hospitalization charges: patients with HAIs versus non-HAI patients. The red solid line represents the median. The blue dashed lines represent the 25th and 75th percentiles (P_25_ and P_75_).

A detailed comparison of hospitalization charges between patients with and without healthcare-associated infections revealed significant differences across multiple cost categories. A violin plot depicting cost divergences is presented in Figure 4. Specifically, median charges were substantially greater in the HAI group for consultation charges (USD 109 vs. USD 17, *p* < 0.001), diagnostic tests charges (USD 407 vs. USD 25, *p* < 0.001), non-pharmacological therapies charges (USD 514 vs. USD 52.10, *p* < 0.001), surgical procedures charges (USD 24 vs. USD 0, *p* < 0.001), laboratory tests charges (USD 1028 vs. USD 100, *p* < 0.001), nursing services charges (USD 161 vs. USD 22, *p* < 0.001), medical supplies charges (USD 236 vs. USD 9, *p* < 0.001), bed charges (USD 174 vs. USD 42, *p* < 0.001), pharmaceuticals (USD 1,858 vs. USD 207, *p* < 0.001), and Chinese Patent Medicines (USD 0.44 vs. USD 0, *p* < 0.001). Consequently, total pharmaceutical charges (including Chinese patent medicine charges and Chinese herbal medicine charges) were also significantly higher in the HAI group (USD 1,875 vs. USD 221, *p* < 0.001). Notably, for Chinese herbal medicine charges and other charges, although the median (IQR) was 0 (0–0) in both groups, the Mann–Whitney U test indicated a statistically significant difference in the overall distribution (*p* < 0.001).

Among the detailed cost categories, pharmaceutical charges exhibited the largest absolute median difference (USD 1650). This was followed by laboratory test charges (USD 928), non-pharmacological therapeutic charges (USD 462), diagnostic test charges (USD 381), medical supply charges (USD 227), nursing service charges (USD 138), bed charges (USD 131), surgical procedure charges (CNY 24), and Chinese patent medicine charges (USD 0.44). The detailed results are presented in Table 3.

### The characteristics of hospitalization charges for different types of HAIs

The five most prevalent types of HAIs in this study were lower respiratory tract infections, bloodstream infections (including central venous catheter-related bloodstream infections, sepsis, and transfusion-associated infections), abdominal and digestive system infections, upper respiratory tract infections, and urinary tract infections.

Hospitalization charges varied across infection types. Pleural Cavity Infection incurred the highest total hospitalization charges, demonstrating the highest median values difference among all infections for pharmaceutical charges. CLABSI was associated with the highest increase in laboratory test charges among all infection categories, with a median rise of USD 1,501 (95%CI: 1131, 2,107) per patient compared to the non-HAIs group.

Compared with the non-HAIs group, patients with lower respiratory tract infections, the most frequent infection type, demonstrated relatively higher median charge differences: USD 1,474 (95% CI, 1230 to 1,626) for laboratory tests, USD 2,491 (95% CI, 2,218 to 2,826) for pharmaceuticals, and USD 9,597 (95% CI, 8,099 to 10,968) for total charges. The detailed data are listed in Table 4.

**Table 4:** Detailed list of major charges for various types of HAIs.

HAIs type	*n*	Laboratory test charges		Pharmaceutical charges		Total hospitalization charges	
		*M* (P_25_~P_75_)	Difference (95% CI)	*M* (P_25_~P_75_)	Difference (95% CI)	*M* (P_25_~P_75_)	Difference (95% CI)
Pleural cavity infection	5	1061.9 (996.0~1933.0)	962.2 (495.46, 2891.78)	3561.3 (2285.8~4349.5)	3354.04 (1451.22, 6130.23)	12151.7 (10588.2~12947.5)	10939.99 (4914.47, 13091.28)
Lower respiratory tract infection	346	1574.3 (776.5~3194.4)	1474.62 (1230.35, 1625.57)	2698.5 (1019.3~7156.5)	2491.33 (2217.87, 2825.95)	10809.2 (4397.8~20002.7)	9597.5 (8099.54, 10968.63)
Surgical site infection	55	1470.6 (346.4~2393.5)	1370.95 (749.18, 1771.60)	1646.1 (452.7~3624.3)	1438.91 (981.57, 2306.41)	10664.9 (3635.3~15922.5)	9453.17 (5799.18, 12229.74)
Central line-associated bloodstream infection	72	1600.9 (798.2~3026.2)	1501.16 (1131.33, 2107.13)	2896.3 (1545.5~7083.6)	2689.09 (2076.26, 3376.18)	10537.6 (5232.3~21923.5)	9325.95 (6410.38, 11543.49)
Gastrointestinal system infection	143	987.3 (436.1~2086.8)	887.57 (716.21, 1179.63)	2128.5 (1016.8~5315.9)	1921.29 (1471.89, 2932.01)	8507.0 (3447.6~15051.0)	7295.34 (5765.49, 9107.73)
Urinary tract infection	114	1022.6 (292.1~1996.2)	922.88 (638.58, 1293.51)	1539.3 (287.8~3645.9)	1332.06 (905.51, 1862.48)	7316.7 (2614.0~13470.5)	6105.06 (3427.82, 8754.01)
Infection of other sites	69	923.2 (413.0~1833.2)	823.47 (483.83, 1076.07)	1624.2 (752.5~4499.3)	1417.02 (976.32, 3055.45)	6518.3 (2304.6~9998.7)	5306.64 (2886.71, 7171.38)
Sepsis	88	1068.0 (576.9~2441.8)	968.32 (787.67, 1174.62)	2133.7 (1072.6~4972.0)	1926.47 (1302.26, 2668.45)	6414.9 (3459.6~11829.6)	5203.23 (3108.65, 7287.61)
Transfusion-transmitted infection	1	979.4 (979.4~979.4)	879.71 (none)	669.9 (669.9~669.9)	462.65 (none)	4353.8 (4353.8~4353.8)	3142.14 (none)
Skin and soft tissue infection	22	635.4 (408.4~1011.7)	535.69 (314.5, 881.59)	1388.3 (647.1~2770.3)	1181.08 (456.32, 2501.95)	3590.6 (2049.3~6950.8)	2378.92 (1033.40, 4622.89)
Upper respiratory tract infection	132	304.9 (119.3~690.6)	205.21 (117.13, 307.58)	304.2 (66.3~860.2)	96.99 (54.29, 268.41)	1270.3 (458.0~3452.3)	58.57 (−419.19, 869.00)
Genital tract infection	22	319.9 (213.5~538.7)	220.24 (118.96, 364.96)	132.9 (61.6~354.7)	−74.35 (145.04, 147.46)	1128.9 (677.3~2214.7)	−82.74 (−498.27, 755.37)

Cost variables for each infection category are expressed as median (25th–75th percentile). The median difference between each infection group and the uninfected reference group and its 95% confidence interval (CI) were calculated using the bootstrap method with 1,000 replications.

## Discussion

Against the backdrop of refined hospital management, infection control is transitioning from an extensive model to intelligent and precise infection prevention. Novel infection control technologies continue to evolve; for instance, micro/nanorobots applied in infection control have emerged (16). At the same time, more comprehensive and sophisticated hospital infection surveillance systems, such as the United States National Healthcare Safety Network (NHSN) has been established. These developments highlight the significant value of infection prevention. However, in practice, due to the public health nature of healthcare-associated infection prevention, the multiplicity of infection risk factors, and the lack of discounting capacity for potential HAI risks in current hospital evaluation systems, the latent value of preventive efforts remains difficult to quantify.

Our research, framed from the perspective of patients as payers, explores methodologies for quantifying HAI-related charges. After conducting 1:1 propensity score matching between infected and non-infected patient groups, it was found that the total hospitalization cost for patients with HAIs was 5.90 times that of non-HAI patients, with a median difference of USD 5,946. In 2024, the per capita GDP in Shenzhen, Guangdong Province, was USD 29,100, which means that the median cost difference accounted for 20.43% of the per capita GDP. This comparative study demonstrates that HAIs, as preventable adverse occurrences, can be mitigated through infection prevention measures, which in turn yield tangible economic returns.

Analysis of the data indicates differences in total hospitalization charges according to the type of healthcare-associated infection. Among these, central line-associated bloodstream infections ranked highest in terms of laboratory charges, as diagnosing these infections typically requires laboratory test results from both peripheral and catheter-drawn blood samples, as well as paired sets of blood cultures from two different sites, making the testing process particularly costly. In this study, the pharmaceutical charges and total hospitalization charges were also elevated, ranking high among all infection categories. This finding is consistent with a meta-analysis on the disease burden of HAIs in United States hospitals (17). Thus, CLABSI is a “high-burden, low-incidence” infection that requires prioritized prevention. Lower respiratory tract infections (346 cases, 32.4%) were the most common, and although the per-case charges difference (USD 10,809) was marginally lower than that of the highest-cost infection type, their high incidence likely leads to a greater aggregate economic burden, thus also warranting close attention. This study also indicated that genital tract infections did not significantly increase hospitalization charges.

To control for confounding variables, we reviewed the existing literature that reported direct medical charges, ensuring that the variables in our study were grounded in established research. We additionally matched patients on the specific time frame of admission, thereby ensuring that the case and control groups were hospitalized under similar temporal conditions. The admitting department was also included as a key matching variable, acknowledging that variations in clinical practice across departments significantly influenced the charges. Furthermore, to avoid immortal time bias, we only included patients whose observation started within three calendar days of admission, using this time point as the starting point for cost calculation, thereby preventing expenses from the first 3 days of hospitalization from affecting the overall study outcomes.

The total charges for the HAI group were significantly higher than those for the non-HAI group, with the largest increases observed in pharmaceutical charges (8.97-fold) and laboratory test charges (10.31-fold). Together, these two categories accounted for approximately 43.42% of the total difference in charges, consistent with previous studies (18). Nursing charges also increased, supporting existing evidence that infection control measures, such as cluster-based care for infected patients, intensify clinical nursing workloads. Although statistically significant differences were observed in other expense categories (e.g., surgical fees, consultation charges, and traditional Chinese medicine), their contribution to the overall cost difference was minimal. In summary, these findings indicate that HAIs primarily drive up charges related to medication and laboratory diagnostics. From a clinical perspective, the management of suspected infections, which involves empirical antibiotic therapy, culture sampling, and subsequent antibiotic adjustment based on susceptibility results, considerably prolongs recovery time and amplifies the economic burden on patients.

While our study adopts the patient perspective in quantifying the burden of HAIs, its findings underscore a critical alignment of interests under China’s DRG payment system. Given that the Diagnosis-Related Groups-Prospective Payment System (DRG-PPS) does not cover the additional costs of HAIs, these expenses are increasingly borne by hospitals under fixed reimbursements. Consequently, effective infection prevention and control transforms from a patient-safety measure into a win-win strategy, safeguarding patient well-being while maintaining hospital fiscal health under stringent cost-containment policies.

Accurate estimation of economic burden is critical for prioritizing and effectively allocating resources. However, this study has limitations in quantifying the burden. First, it is largely confined to direct medical charges from the payer perspective, whereas the burden of healthcare-associated infections extends beyond direct inpatient expenses to include other direct non-medical charges (e.g., reduced time participating in social production due to hospitalization). It also does not account for indirect charges from a societal perspective, such as disease burden (i.e., productivity loss) and mortality burden (i.e., premature death)-related expenses. Furthermore, the single-center design may limit the generalizability of our results, and unmeasured heterogeneity across different wards and specialties could introduce bias. Although PSM was used to balance observed covariates, residual confounding due to unmeasured factors (e.g., disease severity) remains possible. Our analysis is subject to potential temporal bias related to the timing of HAI onset, as the precise attribution of pre- and post-infection costs can be complex; We lacked information on key clinical outcomes such as, ICU transfer, readmission, and need for invasive support, which are crucial for a full assessment of the HAI burden. Finally, the charges analyzed represent nominal payments rather than true accounting costs, which should be considered in economic interpretation.

In summary, this study quantifies the substantial economic burden of HAIs from a patient-payer perspective, identifying significant cost increases. Crucially, because these incremental costs remain unreimbursed under the DRG-PPS, they constitute a direct economic deficit for healthcare providers. This fiscal reality elevates infection prevention from a traditional clinical priority to a fundamental pillar of institutional sustainability. Future research may consider developing standardized and quantifiable metrics through multi-center studies to assess the economic returns on infection prevention investments.

## Conclusion

This study indicates a 5.90-fold increase in total hospitalization charges for HAI patients, driven primarily by surges in laboratory charges (10.31-fold) and Pharmaceutical charges (8.97-fold). CLABSIs presented the highest laboratory charges burden, LRTIs were identified as the most prevalent and demonstrated significantly increased direct medical charges. Therefore, hospital administrators and healthcare providers in China should recognize the current HAI situation and work to strengthen their management.

## Data Availability

The data analyzed in this study is subject to the following licenses/restrictions: only for use in the hospital infection management research of the Seventh Affiliated Hospital of Sun Yat-sen University. Requests to access these datasets should be directed to xinhaizhao.zxh1992zt@hotmail.com.
